# Supplementary material for: Effects of Global Climate Warming on the Biological Characteristics of Spodoptera frugiperda (J.E. Smith) (Lepidoptera: Noctuidae)
Source: Insects. 2024 Sep 12;15(9):689. doi: 10.3390/insects15090689 (PMC11432313; doi:10.3390/insects15090689)
Supplement: Supplementary file 1 [file insects-15-00689-s001.zip › insects-3186819-Supplementary.pdf]

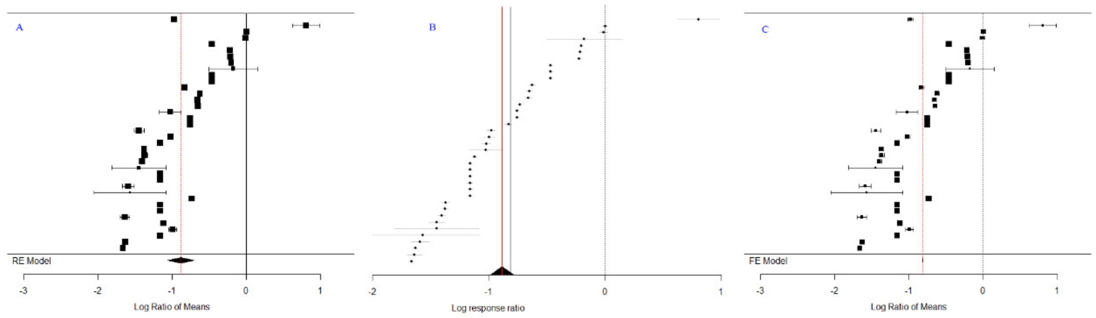

Figure S1 Comparison of effect sizes between random-effects and fixed-effects models (Panel A displays the results of the random-effects model calculation, E = -0.8841, 95% confidence interval ranging from -1.0609 to -0.7073, Q (df = 37) = 51662.5037, $p < 0.0001$, $I^2 = 99.95\%$. Panel C shows the results of the fixed-effects model calculation, E = -0.8597, 95% confidence interval ranging from -0.8612 to -0.8562, Q (df = 37) = 51662.5037, $p < 0.0001$, $I^2 = 99.75\%$. Panel B compares the results between random-effects and fixed-effects models. The calculations indicate that, under both random-effects and fixed-effects modeling conditions, the duration of the egg stage of the *S. Frugiperda* significantly decreases with increasing temperature. Black squares represent the values of the cumulative effect size for each variable. Black horizontal lines represent the 95% confidence interval. In Figure A and C, the red solid lines indicate the cumulative effect values. In Figure B, the red solid line represents the cumulative effect values calculated using a random effects model.)

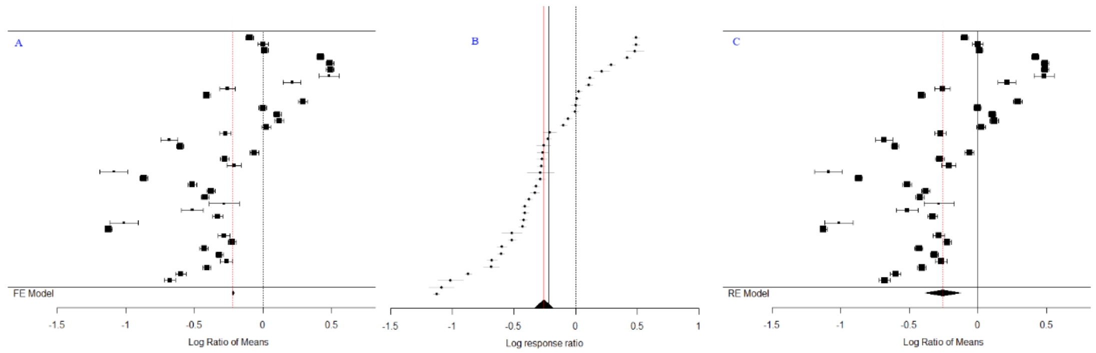

Figure S2 Comparison of effect sizes between random-effects and fixed-effects models (Panel A displays the results of the random-effects model calculation, E = -0.2560, 95% confidence interval ranging from -0.3857 to -0.1263, Q (df = 38) = 21034.5417, $p < 0.0001$, $I^2 = 99.82\%$. Panel C shows the results of the fixed-effects model calculation, E = -0.2190, 95% confidence interval ranging from -0.2245 to -0.2136, Q (df = 38) = 21034.5417, $p < 0.0001$, $I^2 = 99.82\%$. Panel B compares the results between random-effects and fixed-effects models. The calculations indicate that, under both random-effects and fixed-effects modeling conditions, the duration of development from egg to adult stage of the *S. Frugiperda* significantly decreases with increasing temperature. Black squares represent the values of the cumulative effect size for each variable. Black horizontal lines represent the 95% confidence interval. In Figure A and C, the red solid lines indicate the cumulative effect values. In Figure B, the red solid line represents the cumulative effect values calculated using a random effects model.)

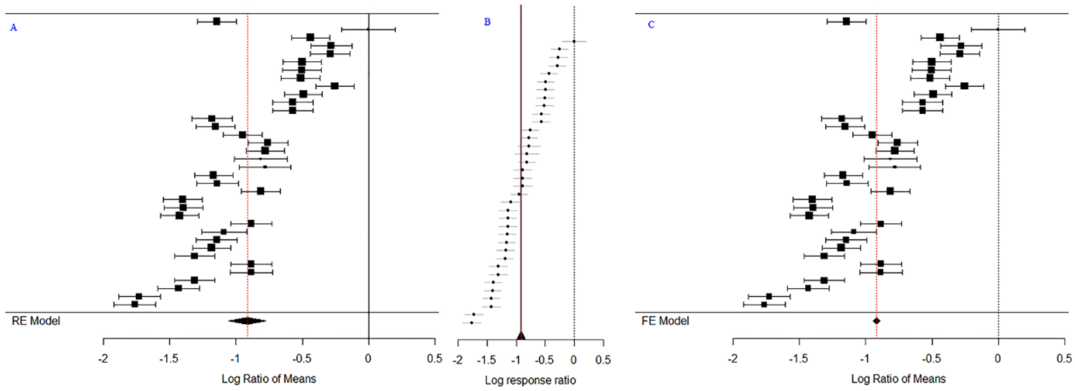

Figure S3 Comparison of effect sizes between random-effects and fixed-effects models (Panel A displays the results of the random-effects model calculation, E = -0.9155, 95% confidence interval ranging from -1.0556 to -0.7754, Q (df = 35) = 1014.9817, p < 0.0001, I^2 = 96.66%. Panel C shows the results of the fixed-effects model calculation, E = -0.9199, 95% confidence interval ranging from -0.9455 to -0.8943, Q (df = 35) = 1014.9817, p < 0.0001, I^2 = 96.55%. Panel B compares the results between random-effects and fixed-effects models. The calculations indicate that, under both random-effects and fixed-effects modeling conditions, the duration of the first instar stage of the *S. Frugiperda* significantly decreases with increasing temperature. Black squares represent the values of the cumulative effect size for each variable. Black horizontal lines represent the 95% confidence interval. In Figure A and C, the red solid lines indicate the cumulative effect values. In Figure B, the red solid line represents the cumulative effect values calculated using a random effects model.)

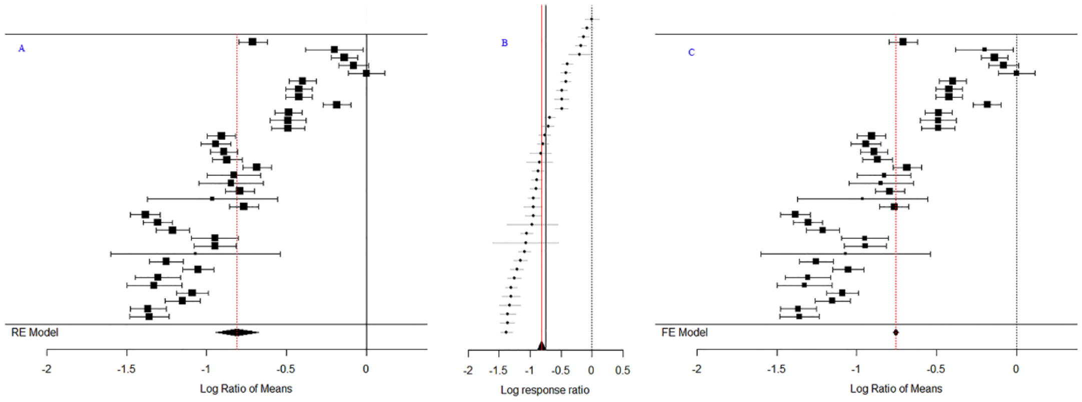

Figure S4 Comparison of effect sizes between random-effects and fixed-effects models (Panel A displays the results of the random-effects model calculation, E = -0.8096, 95% confidence interval ranging from -0.9425 to -0.6766, Q (df = 35) = 2035.6998, $p < 0.0001$, $I^2 = 98.26\%$. Panel C shows the results of the fixed-effects model calculation, E =-0.7538, 95% confidence interval ranging from -0.7711 to -0.7365, Q (df = 35) = 2035.6998, $p < 0.0001$, $I^2 = 98.28\%$. Panel B compares the results between random-effects and fixed-effects models. The calculations indicate that, under both random-effects and fixed-effects modeling conditions, the duration of the second instar stage of the *S. Frugiperda* significantly decreases with increasing temperature. Black squares represent the values of the cumulative effect size for each variable. Black horizontal lines represent the 95% confidence interval. In Figure A and C, the red solid lines indicate the cumulative effect values. In Figure B, the red solid line represents the cumulative effect values calculated using a random effects model.)

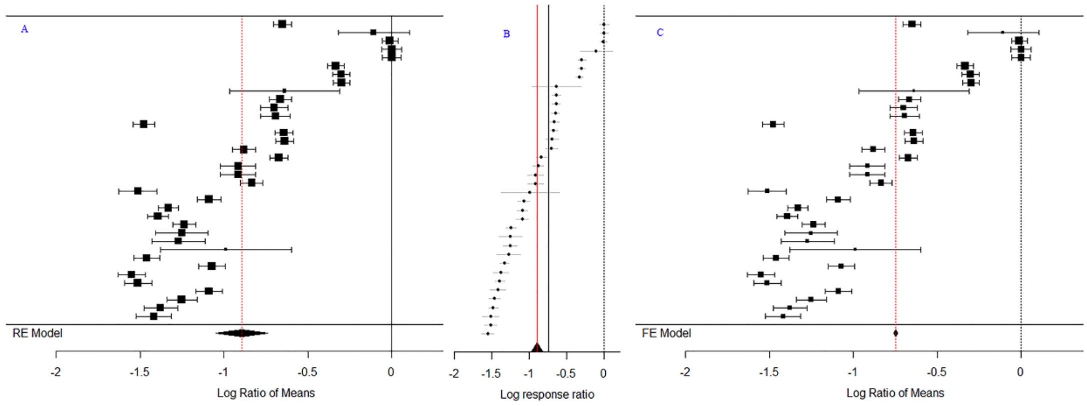

Figure S5 Comparison of effect sizes between random-effects and fixed-effects models (Panel A displays the results of the random-effects model calculation, E = -0.8946, 95% confidence interval ranging from -1.0507 to -0.7386, Q (df = 35) = 6709.9338, p < 0.0001, I^2 =99.42%. Panel C shows the results of the fixed-effects model calculation, E = -0.7477, 95% confidence interval ranging from -0.7595 to -0.7360, Q (df = 35) = 2035.6998, p < 0.0001, I^2 = 99.48%. Panel B compares the results between random-effects and fixed-effects models. The calculations indicate that, under both random-effects and fixed-effects modeling conditions, the duration of the third instar stage of the *S. Frugiperda* significantly decreases with increasing temperature. Black squares represent the values of the cumulative effect size for each variable. Black horizontal lines represent the 95% confidence interval. In Figure A and C, the red solid lines indicate the cumulative effect values. In Figure B, the red solid line represents the cumulative effect values calculated using a random effects model.)

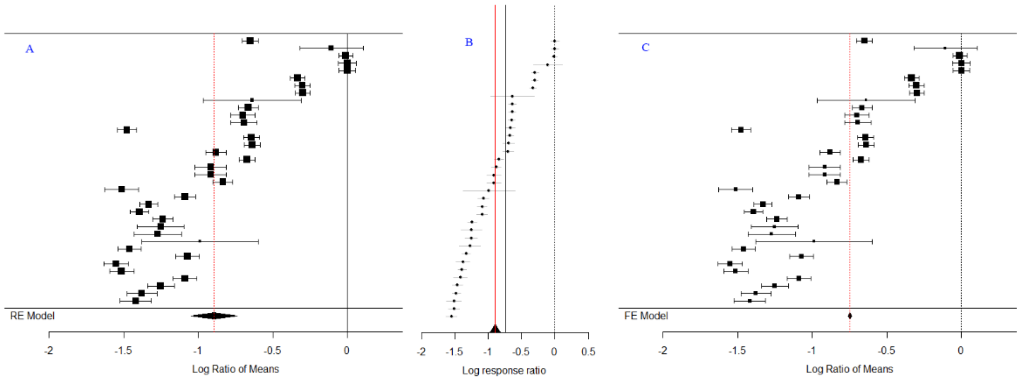

Figure S6 Comparison of effect sizes between random-effects and fixed-effects models (Panel A displays the results of the random-effects model calculation, E =-0.8952, 95% confidence interval ranging from -1.0476 to -0.7427, Q (df = 35) = 13509.9649, $p < 0.0001$, $I^2 = 99.72\%$. Panel C shows the results of the fixed-effects model calculation, E = -0.7477, 95% confidence interval ranging from -0.7595 to -0.7360, Q (df = 35) = 13509.9649, $p < 0.0001$, $I^2 = 99.68\%$. Panel B compares the results between random-effects and fixed-effects models. The calculations indicate that, under both random-effects and fixed-effects modeling conditions, the duration of the fourth instar stage of the *S. Frugiperda* significantly decreases with increasing temperature. Black squares represent the values of the cumulative effect size for each variable. Black horizontal lines represent the 95% confidence interval. In Figure A and C, the red solid lines indicate the cumulative effect values. In Figure B, the red solid line represents the cumulative effect values calculated using a random effects model.)

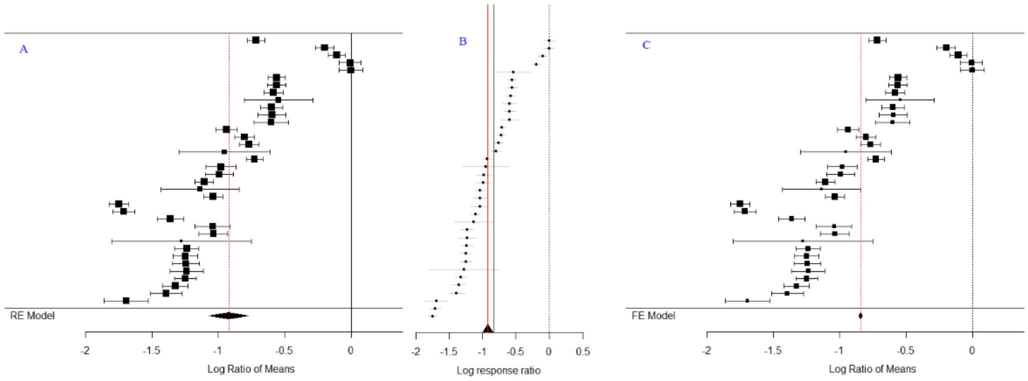

Figure S7 Comparison of effect sizes between random-effects and fixed-effects models (Panel A displays the results of the random-effects model calculation, E = -0.9214, 95% confidence interval ranging from -1.0697 to -0.7732, Q (df = 35) = 3728.5542, p < 0.0001, I^2 = 98.98%. Panel C shows the results of the fixed-effects model calculation, E = -0.8467, 95% confidence interval ranging from -0.8505 to -0.8350, Q (df = 35) = 3728.5542, p < 0.0001, I^2 = 99.68%. Panel B compares the results between random-effects and fixed-effects models. The calculations indicate that, under both random-effects and fixed-effects modeling conditions, the duration of the fifth instar stage of the *S. Frugiperda* significantly decreases with increasing temperature. Black squares represent the values of the cumulative effect size for each variable. Black horizontal lines represent the 95% confidence interval. In Figure A and C, the red solid lines indicate the cumulative effect values. In Figure B, the red solid line represents the cumulative effect values calculated using a random effects model.)

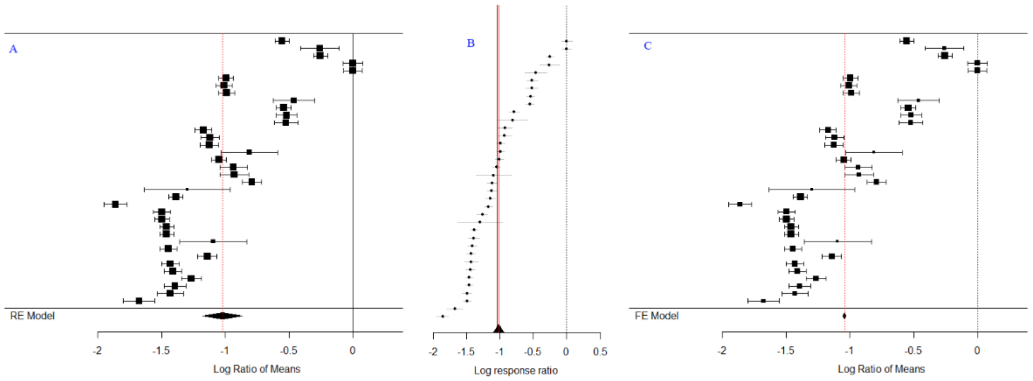

Figure S8 Comparison of effect sizes between random-effects and fixed-effects models (Panel A displays the results of the random-effects model calculation, E = -1.0217, 95% confidence interval ranging from -1.1758 to -0.8677, Q (df = 35) = 5289.4684, $p < 0.0001$, $I^2 = 99.36\%$. Panel C shows the results of the fixed-effects model calculation, E =-1.0424, 95% confidence interval ranging from -1.0549 to -1.0301, Q (df = 35) = 5289.4684, $p < 0.0001$, $I^2 = 99.34\%$. Panel B compares the results between random-effects and fixed-effects models. The calculations indicate that, under both random-effects and fixed-effects modeling conditions, the duration of the sixth instar stage of the *S. Frugiperda* significantly decreases with increasing temperature. Black squares represent the values of the cumulative effect size for each variable. Black horizontal lines represent the 95% confidence interval. In Figure A and C, the red solid lines indicate the cumulative effect values. In Figure B, the red solid line represents the cumulative effect values calculated using a random effects model.)

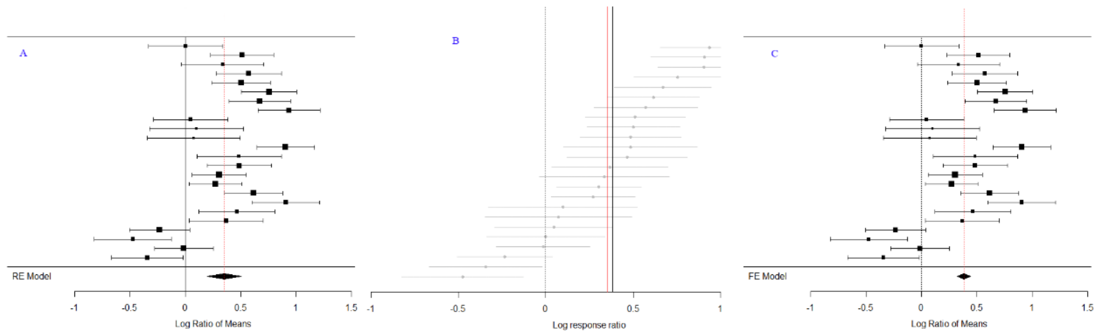

Figure 9S Comparison of effect sizes between random-effects and fixed-effects models (Panel A displays the results of the random-effects model calculation. Panel C shows the results of the fixed-effects model calculation, E = -0.2190, 95% confidence interval ranging from -0.2245 to -0.2136, Q (df = 38) = 21034.5417, $p < 0.0001$, I^2 = 99.84%. Panel B compares the results between random-effects and fixed-effects models. The calculations indicate that, under both random-effects and fixed-effects modeling conditions, the oviposition rate of female *S. Frugiperda* significantly increases with increasing temperature. Black squares represent the values of the cumulative effect size for each variable. Black horizontal lines represent the 95% confidence interval. In Figure A and C, the red solid lines indicate the cumulative effect values. In Figure B, the red solid line represents the cumulative effect values calculated using a random effects model.)

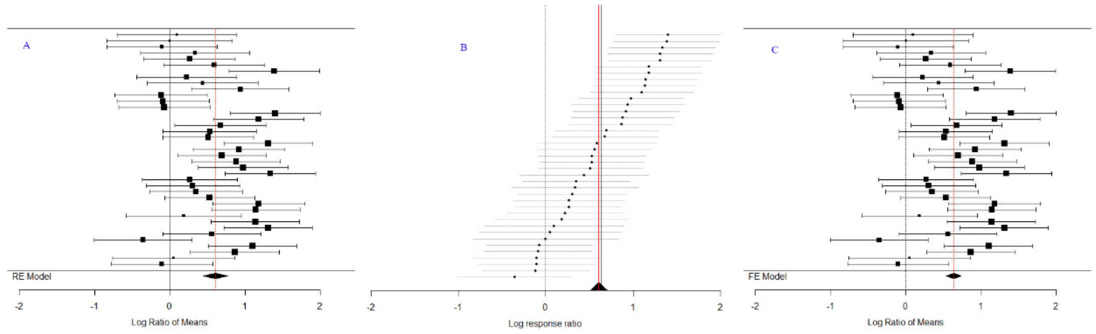

Figure S10 Comparison of effect sizes between random-effects and fixed-effects models (Panel A displays the results of the random-effects model calculation, E =0.6075, 95% confidence interval ranging from 0.4465 to 0.7678, Q (df = 38) = 94.5784, p < 0.0001, I^2 = 59.85%. Panel C shows the results of the fixed-effects model calculation, E = 0.6353, 95% confidence interval ranging from 0.5340 to 0.7367, Q (df = 38) = 94.5784, p < 0.0001, I^2 = 59.82%. Panel B compares the results between random-effects and fixed-effects models. The calculations indicate that, under both random-effects and fixed-effects modeling conditions, the duration of the oviposition period of female *S. Frugiperda* significantly increases with increasing temperature. Black squares represent the values of the cumulative effect size for each variable. Black horizontal lines represent the 95% confidence interval. In Figure A and C, the red solid lines indicate the cumulative effect values. In Figure B, the red solid line represents the cumulative effect values calculated using a random effects model.)

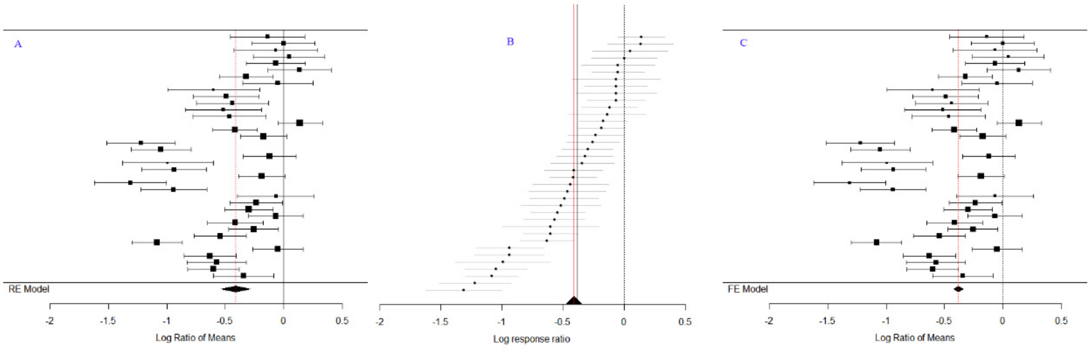

Figure S11 Comparison of effect sizes between random-effects and fixed-effects models (Panel A displays the results of the random-effects model calculation, E = -0.4103, 95% confidence interval ranging from -0.5345 to -0.2861, Q (df = 36) = 298.3982, p < 0.0001, I^2 = 88.79%. Panel C shows the results of the fixed-effects model calculation, E = -0.3816, 95% confidence interval ranging from -0.4228 to -0.3404, Q(df = 38) = 298.3982, p < 0.0001, I^2 = 87.94%. Panel B compares the results between random-effects and fixed-effects models. The calculations indicate that, under both random-effects and fixed-effects modeling conditions, the duration of the pre-oviposition period of female *S. Frugiperda* significantly decreases with increasing temperature. Black squares represent the values of the cumulative effect size for each variable. Black horizontal lines represent the 95% confidence interval. In Figure A and C, the red solid lines indicate the cumulative effect values. In Figure B, the red solid line represents the cumulative effect values calculated using a random effects model.)

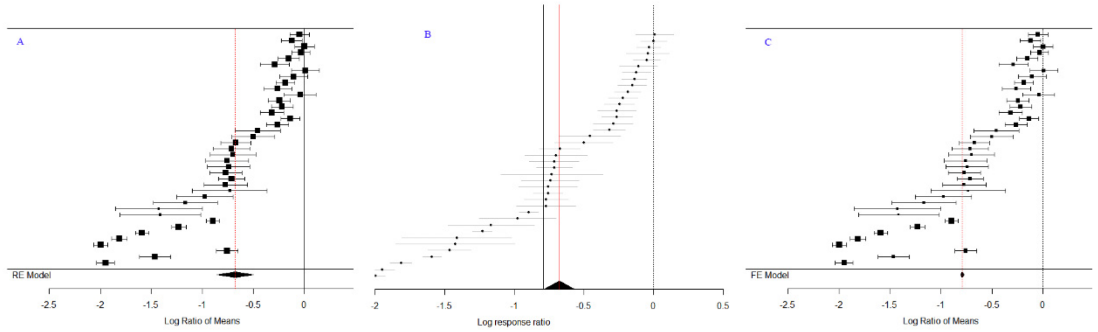

Figure S12 Comparison of effect sizes between random-effects and fixed-effects models (Panel A displays the results of the random-effects model calculation, E =-0.6780, 95% confidence interval ranging from -0.8597 to -0.4963, Q (df = 38) = 5646.9218, p < 0.0001, I^2 = 98.97%. Panel C shows the results of the fixed-effects model calculation, E = -0.6501, 95% confidence interval ranging from -0.6582 to -0.6519, Q (df = 38) = 5646.9218, p < 0.0001, I^2 = 99.33%. Panel B compares the results between random-effects and fixed-effects models. The calculations indicate that, under both random-effects and fixed-effects modeling conditions, the duration of the pupal stage of the *S. Frugiperda* significantly decreases with increasing temperature. Black squares represent the values of the cumulative effect size for each variable. Black horizontal lines represent the 95% confidence interval. In Figure A and C, the red solid lines indicate the cumulative effect values. In Figure B, the red solid line represents the cumulative effect values calculated using a random effects model.)

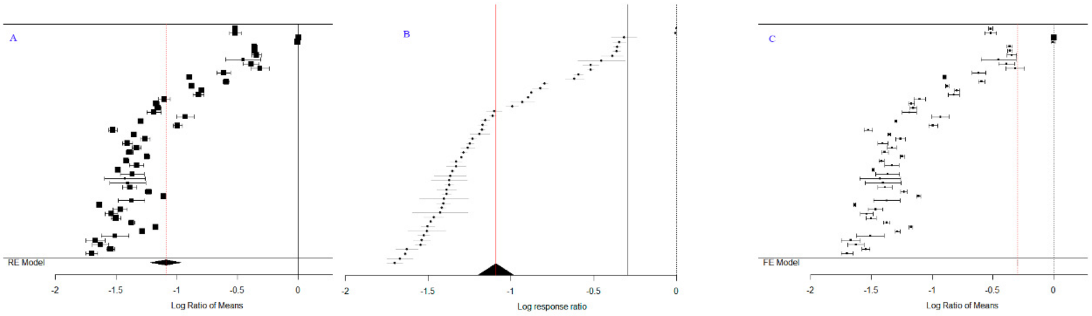

Figure S13 Comparison of effect sizes between random-effects and fixed-effects models (Panel A displays the results of the random-effects model calculation, E = -0.7901, 95% confidence interval ranging from -0.8082 to -0.7719, Q (df = 38) = 6636.9817, $p < 0.0001$, $I^2 = 99.33\%$. Panel C shows the results of the fixed-effects model calculation. Panel B compares the results between random-effects and fixed-effects models. The calculations indicate that, under both random-effects and fixed-effects modeling conditions, the duration of the pupal stage of the *S. Frugiperda* significantly decreases with increasing temperature. Black squares represent the values of the cumulative effect size for each variable. Black horizontal lines represent the 95% confidence interval. In Figure A and C, the red solid lines indicate the cumulative effect values. In Figure B, the red solid line represents the cumulative effect values calculated using a random effects model.)

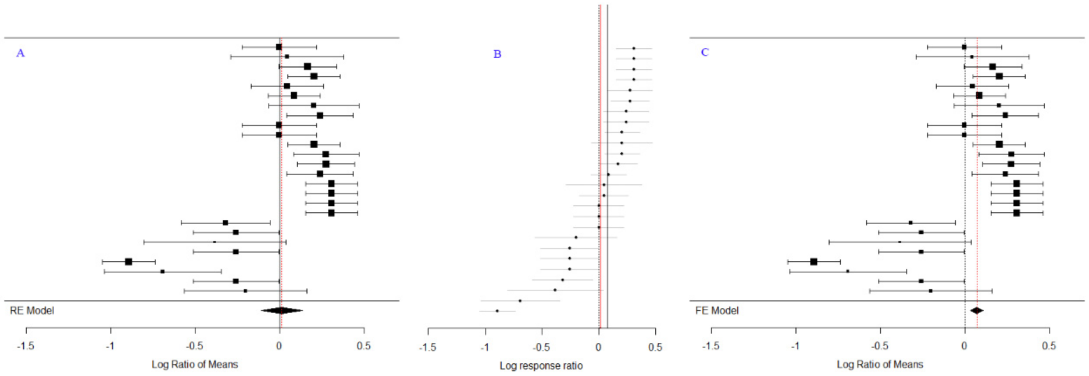

Figure S14 Comparison of effect sizes between random-effects and fixed-effects models (Panel A displays the results of the random-effects model calculation, E = 0.0134, 95% confidence interval ranging from -0.1098 to 0.1365, Q (df = 25) = 262.5494, p < 0.0001, I^2 =89.72%. Panel C shows the results of the fixed-effects model calculation, E = 0.0724, 95% confidence interval ranging from 0.0338 to 0.1111, Q (df = 25) = 262.5494, p < 0.0001, I^2 =90.48%. Panel B compares the results between random-effects and fixed-effects models. The calculations indicate that, under both random-effects and fixed-effects modeling conditions, there is no significant change in the eclosion rate of *S. Frugiperda* pupae with increasing temperature. Black squares represent the values of the cumulative effect size for each variable. Black horizontal lines represent the 95% confidence interval. In Figure A and C, the red solid lines indicate the cumulative effect values. In Figure B, the red solid line represents the cumulative effect values calculated using a random effects model.)

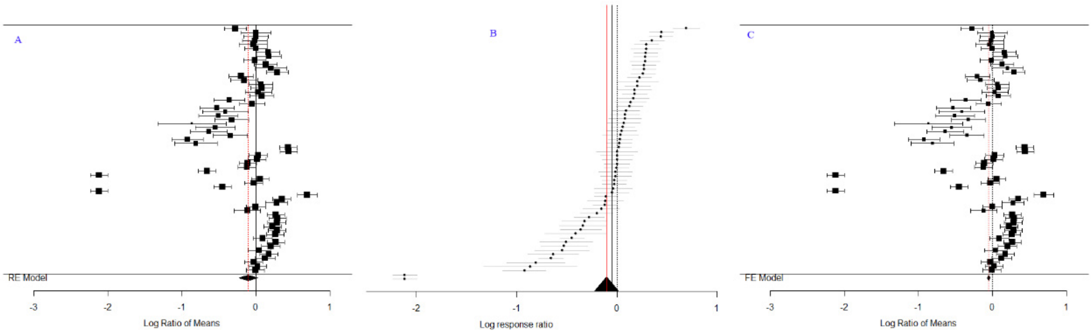

Figure S15 Comparison of effect sizes between random-effects and fixed-effects models (Panel A displays the results of the random-effects model calculation, E =-0.1016, 95% confidence interval ranging from -0.2252 to 0.1365, Q (df = 61) = 3246.3144, $p < 0.0001$, $I^2 = 97.75\%$. Panel C shows the results of the fixed-effects model calculation, E = -0.0506, 95% confidence interval ranging from -0.0690 to -0.0322, Q (df = 61) = 3246.3144, $p < 0.0001$, $I^2 = 98.12\%$. Panel B compares the results between random-effects and fixed-effects models. The calculations indicate that, under both random-effects and fixed-effects modeling conditions, the lifespan of adult *S. Frugiperda* significantly increases with increasing temperature. Black squares represent the values of the cumulative effect size for each variable. Black horizontal lines represent the 95% confidence interval. In Figure A and C, the red solid lines indicate the cumulative effect values. In Figure B, the red solid line represents the cumulative effect values calculated using a random effects model.)

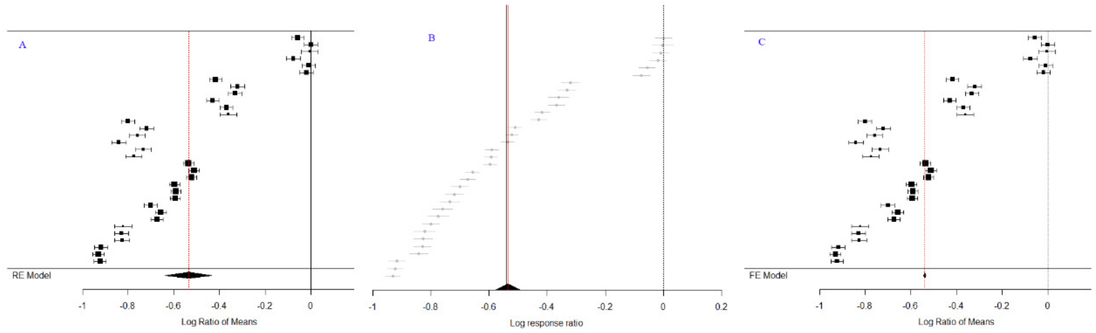

Figure S16 Comparison of effect sizes between random-effects and fixed-effects models (Panel A displays the results of the random-effects model calculation, E = -0.5349, 95% confidence interval ranging from -0.6366 to -0.4331, Q (df = 32) = 12303.1473, p < 0.0001, I^2 = 99.77%. Panel C shows the results of the fixed-effects model calculation, E = -0.5405, 95% confidence interval ranging from -0.5453 to -0.5356, Q (df = 61) = 12303.1473, p < 0.0001, I^2 = 99.74%. Panel B compares the results between random-effects and fixed-effects models. The calculations indicate that, under both random-effects and fixed-effects modeling conditions, the lifespan of *S. Frugiperda* significantly decreases with increasing temperature. Black squares represent the values of the cumulative effect size for each variable. Black horizontal lines represent the 95% confidence interval. In Figure A and C, the red solid lines indicate the cumulative effect values. In Figure B, the red solid line represents the cumulative effect values calculated using a random effects model.)
